# Physiological and skeletal muscle responses to high-intensity interval exercise in Thoroughbred horses

**DOI:** 10.3389/fvets.2023.1241266

**Published:** 2023-11-09

**Authors:** Kazutaka Mukai, Hajime Ohmura, Yuji Takahashi, Yusaku Ebisuda, Koki Yoneda, Hirofumi Miyata

**Affiliations:** ^1^Sports Science Division, Equine Research Institute, Japan Racing Association, Shimotsuke, Japan; ^2^Biological Sciences, Graduate School of Sciences and Technology for Innovation, Yamaguchi University, Yamaguchi, Japan

**Keywords:** high-intensity interval training, skeletal muscle, horse, mitochondria, lactate

## Abstract

**Introduction:**

The purpose of this study was to determine whether acute high-intensity interval exercise or sprint interval exercise induces greater physiological and skeletal muscle responses compared to moderate-intensity continuous exercise in horses.

**Methods:**

In a randomized crossover design, eight trained Thoroughbred horses performed three treadmill exercise protocols consisting of moderate-intensity continuous exercise (6 min at 70% VO_2_max; MICT), high-intensity interval exercise (6 × 30 s at 100% VO_2_max; HIIT), and sprint interval exercise (6 × 15 s at 120% VO_2_max; SIT). Arterial blood samples were collected to measure blood gas variables and plasma lactate concentration. Biopsy samples were obtained from the gluteus medius muscle before, immediately after, 4 h, and 24 h after exercise for biochemical analysis, western blotting and real-time RT-PCR. Effects of time and exercise protocol were analyzed using mixed models (*p* < 0.05).

**Results:**

Heart rate and plasma lactate concentration at the end of exercise were higher in HIIT and SIT than those in MICT (heart rate, HIIT vs. MICT, *p* = 0.0005; SIT vs. MICT, *p* = 0.0015; lactate, HIIT vs. MICT, *p* = 0.0014; SIT vs. MICT, *p* = 0.0003). Arterial O_2_ saturation and arterial pH in HIIT and SIT were lower compared with MICT (SaO_2_, HIIT vs. MICT, *p* = 0.0035; SIT vs. MICT, *p* = 0.0265; pH, HIIT vs. MICT, *p* = 0.0011; SIT vs. MICT, *p* = 0.0023). Muscle glycogen content decreased significantly in HIIT (*p* = 0.0004) and SIT (*p* = 0.0016) immediately after exercise, but not in MICT (*p* = 0.19). Phosphorylation of AMP-activated protein kinase (AMPK) in HIIT showed a significant increase immediately after exercise (*p* = 0.014), but the increase was not significant in MICT (*p* = 0.13) and SIT (*p* = 0.39). At 4 h after exercise, peroxisome proliferator-activated receptor γ co-activator-1α mRNA increased in HIIT (*p* = 0.0027) and SIT (*p* = 0.0019) and vascular endothelial growth factor mRNA increased in SIT (*p* = 0.0002).

**Discussion:**

Despite an equal run distance, HIIT and SIT cause more severe arterial hypoxemia and lactic acidosis compared with MICT. In addition, HIIT activates the AMPK signaling cascade, and HIIT and SIT elevate mitochondrial biogenesis and angiogenesis, whereas MICT did not induce any significant changes to these signaling pathways.

## 1. Introduction

High-intensity interval training (HIIT) has gained popularity recently in human athletes and is defined as intermittent periods of intense exercise, in which near maximal efforts are performed at an intensity that elicits >80% of maximal oxygen consumption (VO_2_max) or heart rate, separated by recovery periods (1). Sprint interval training (SIT) is characterized by efforts performed at an intensity that elicits >100% VO_2_max, including all-out or supramaximal efforts (1). When estimated energy expenditure is equivalent, these types of interval training have reported to induce similar or greater physiological adaptations, including exercise performance, aerobic capacity, and cardiovascular function, compared to moderate-intensity continuous training (MICT) with less time commitment in humans (1–4). For example, after 8 weeks of work-matched MICT and HIIT in human subjects, HIIT showed improvements in maximal cardiac output, VO_2_ kinetics and skeletal muscle mitochondrial respiration whereas MICT did not (2). Six sessions of SIT for 2 weeks increased cycle endurance capacity, muscle glycogen content and mitochondrial enzyme activity in humans (5).

Improvements in aerobic capacity and energy aerobic metabolism during exercise are mainly related with central adaptations, including increased maximal cardiac output, maximal stroke volume and blood volume, and peripheral adaptations such as increased skeletal muscle capillary density and mitochondrial contents in humans (6, 7) and horses (8–10). Exercise training increases mitochondrial density and, as a result, decreases glycogen degradation and lactate accumulation at a given exercise intensity, allowing individuals to exercise for longer durations and at greater percentages of their VO_2_max (11). Several reports have demonstrated that HIIT increases the maximal activity of citrate synthase (CS) and cytochrome c oxidase (COX), which reflects increased skeletal muscle mitochondrial content in rodents (12) and humans (13–15). Mitochondrial biogenesis appears to result from the cumulative effects of transient increases in mRNA encoding mitochondrial proteins, such as peroxisome proliferator-activated receptor γ co-activator (PGC)-1α, CS and COX IV, after exercise sessions (16). Previous work has shown that these pathways are regulated by PGC-1α (17). PGC-1α is a transcriptional co-activator that regulates genes associated with energy metabolism and is the master regulator of mitochondrial biogenesis (18). Skeletal muscle-specific PGC-1α knock-out mice have reduced endurance capacity and showed a shift from oxidative type I and IIa fibers toward fast-twitch glycolytic type IIx and IIb fibers (19). Furthermore, the upstream signals that activate PGC-1α and mitochondrial biogenesis in response to high-intensity interval exercise appear to be AMP-activated protein kinase (AMPK) and p38 mitogen-activated protein kinase (MAPK) (20). Capillaries are essential to deliver O_2_ to mitochondria to produce ATP, and exercise training enhances angiogenesis and increases capillary density that controls the rate of oxygen delivery in skeletal muscle. Vascular endothelial growth factor (VEGF) is a master regulator of angiogenesis. Several human and equine studies have evaluated changes associated with post-exercise angiogenesis and have shown that high intensity exercise induces angiogenesis (10, 21, 22).

Thoroughbred horses have a high exercise performance, aerobic capacity, and skeletal muscle mass. In a 1,200 m race, the peak heart rate and blood lactate concentration of horses exceeds 210 bpm and 20 mmoL/L, respectively (23). To compete in Thoroughbred races, horses need to train at high-intensity similar to races and adapt to these mechanical and metabolic stimuli that they experience in races. Furthermore, over 80% of the middle gluteal muscle consists of type IIa and IIx fibers in Thoroughbred horses (24), and therefore, high-intensity exercise is essential for training adaptations to stimulate these fast-twitch muscle fibers. In addition, previous human studies demonstrated that mitochondrial adaptations may occur in a fiber type-dependent manner (25, 26). For example, the activation of AMPK induced by HIIT was greater in type II fibers than type I fibers (25), and type IIa fibers showed a greater increase in mitochondrial volume than type I fibers after 6 weeks of endurance training (26). These findings suggest that the difference in skeletal muscle properties between humans and horses would induce differentiated responses in mitochondrial biogenesis. On the other hand, horses suffer various extents of musculoskeletal injuries, including fracture, osteoarthritis, superficial digital flexor tendon and suspensory ligament injury, and rhabdomyolysis, as a result of training and races, which leads to cessation of training or reduced training intensity and/or volume. Therefore, it is essential for Thoroughbred racehorses to balance high-intensity training and musculoskeletal injury prevention. Our unpublished logistic analysis indicate that the longer daily exercise distance of cantering at moderate-intensity (6.7–13.3 m/s) increased the risk of fracture and superficial digital flexor tendinitis (27). We considered that HIIT and/or SIT consisted of galloping at high-intensity and trotting, without cantering at moderate-intensity, would concurrently reduce the risk of fracture and tendon injury and induce superior training adaptations that enhance exercise performance, aerobic capacity and skeletal muscle metabolism compared to MICT. Therefore, we assumed that HIIT would be a potential strategy for efficient training for racehorses. However, there are only a few published studies on physiological and skeletal muscle responses to HIIT and SIT in Thoroughbred horses (28, 29).

The aim of the present study was to compare physiological and skeletal muscle responses after a single bout of MICT, HIIT or SIT matched for total run distance in a crossover design. Arterial blood samples were collected during exercise for blood gas analysis and muscle biopsy samples were obtained before and after exercise to determine glycogen depletion and the phosphorylation and the mRNA expression of target proteins. We hypothesized that HIIT and SIT would induce greater physiological and skeletal muscle responses compared to MICT in Thoroughbred horses.

## 2. Materials and methods

### 2.1. Horses

Eight untrained healthy Thoroughbreds (4 geldings, 4 females; mean ± SE age, 6.0 ± 0.8 years; body weight, 505 ± 18 kg; VO_2_max, 172 ± 5 mL/kg/min; speed eliciting 100% VO_2_max, 11.3 ± 0.2 m/s at the onset of the study) were used in this study. The horses had a carotid artery surgically moved from the carotid sheath to a subcutaneous location under sevoflurane anesthesia to facilitate arterial catheterization. At least 12 months after surgery, the horses were trained to run on a treadmill (Sato I, Sato AB, Uppsala, Sweden) while wearing an open-flow mask (30). The horses were trained for 2 days/week on a treadmill at a 6% incline and were kept in a 17 × 22 m yard for approximately 6 h/day on the other 5 days for 4 weeks before preliminary incremental exercise tests began. All horses received 1 kg of oats, 1 kg of pelleted feed, and 3 kg of timothy hay in the morning and 1 kg of oats, 2 kg of pelleted feed (Power up horse II, Nosan Corporation, Yokohama, Japan), and 3 kg of timothy hay in the afternoon. Water was available *ad libitum* during the study.

Before the onset of the study, visual lameness and physical examination, including cardiac auscultation and ECG analysis, were conducted by experienced veterinarians to rule out clinical disorders and exercise intolerance. Additionally, at least two experienced veterinarians carried out clinical examination before training and exercise tests throughout the study. When horses showed lameness or other clinical disorders, they were excluded from the study.

### 2.2. Experimental design

In a randomized crossover design, the horses performed three exercise protocols consisting of MICT (6 min at 70% VO_2_max), HIIT (6 × [30 s at 100% VO_2_max with 30 s recovery at 30% VO_2_max]), and SIT (6 × [15 s at 120% VO_2_max with 70 s recovery at 30% VO_2_max]) on a treadmill inclined at 6%. Each exercise session was separated by 6 days to ensure a sufficient washout interval. The horses walked for 1 h/day in a walker without treadmill exercise and were pastured in a yard during the washout interval.

### 2.3. Preliminary incremental exercise tests

Incremental exercise tests (IET) were conducted one week before the onset of the experiment to measure VO_2_max and 100% VO_2_max. The procedure for the IET for oxygen consumption measurements has been described previously (31). Briefly, after warm-up at 4 m/s for 3 min, the horse exercised for 2 min each at 1.7, 4, 6, 8, 10, 12, and 13 m/s on a 6% inclined treadmill until the horse could not maintain its position with humane encouragement. Horses wore an open-flow mask on the treadmill through which a rheostat-controlled blower drew air. Air flowed through 25 cm diameter tubing and across a pneumotachograph (LF-150B, Vise Medical, Chiba, Japan) connected to a differential pressure transducer (TF-5, Vise Medical, Chiba, Japan). Oxygen and CO_2_ concentrations were measured with an O_2_ and CO_2_ analyzer (MG-360, Vise Medical, Chiba, Japan), and calibrations were used to calculate rates of O_2_ consumption and CO_2_ production with mass flow meters (CR-300, Kofloc, Kyoto, Japan) using the N_2_-dilution/CO_2_-addition mass-balance technique (32). Gas analyzer and mass flowmeter outputs for the final 30 s of each step were also recorded on personal computers using commercial hardware and software (DI-720 and Windaq Pro+, DATAQ, Akron, OH) with sampling at 200 Hz.

### 2.4. Arterial blood sampling and heart rate measurements

Before leading a horse onto the treadmill, an 18-gauge catheter (Surflow, Terumo, Tokyo, Japan) was placed in the horse’s left carotid artery, and an 8-F introducer (MO95H-8, Baxter International, Deerfield, IL) was placed in the right jugular vein. A Swan-Ganz catheter (SP5107U, Becton, Dickinson and Company, Franklin Lakes, NJ) was passed via the right jugular vein so that its tip was positioned in the pulmonary artery, confirmed by measuring pressure at its tip with a pressure transducer (P23XL, Becton, Dickinson and Company, Franklin Lakes, NJ). Arterial blood samples from the 18-gauge carotid catheter were collected into heparinized syringes every 2 min in MICT, the final 10 s of each exercise in HIIT and SIT, and at 1, 3, and 5 min after the exercise. Blood samples were stored on ice immediately after collection. Blood samples were analyzed with a blood gas analyzer (ABL800 FLEX, Radiometer, Copenhagen, Denmark) and for O_2_ saturation (SaO_2_) with a hemoximeter (ABL80 FLEX-CO-OX, Radiometer, Copenhagen, Denmark). Following measurement of blood gasses and oximetry, the blood was centrifuged at 1740 × g for 10 min, followed by measurement of plasma lactate concentration using a lactate analyzer (Biosen S-Line, EKF-diagnostic GmbH, Barleben, Germany). The Swan-Ganz catheter in the pulmonary artery was connected to a cardiac output computer (COM-2, Baxter International, Deerfield, IL) to measure pulmonary arterial temperature (T_PA_), which was recorded at each blood sampling and used to correct the blood gas measurements. Heart rate was recorded using a commercial heart rate monitor (S810, Polar, Kempele, Finland) and mean heart rate was calculated for the final 10 s of each timing. The horse’s coat, where the electrodes of the heart rate monitor were placed, was soaked with water. One of the electrodes of the heart rate monitor was attached to the saddle blanket and was placed on the left side of the horse 10 cm below the withers. The other electrode was attached to the elastic girth and was placed on the horse at the level of the left elbow. Then, the transmitter and the receiver of the heart rate monitor were also attached to the saddle blanket.

### 2.5. Muscle sampling

Muscle biopsy sampling site was set at one-third of the distance from the coxal tuber on an imaginary line drawn from the coxal tuber to the root of the tail. After shaved and aseptically prepared, the skin was locally anesthetized by subcutaneous injection of 0.5 mL of 2% lidocaine (Sandoz K.K., Tokyo, Japan). Skin incisions were made by scalpel No. 11 (Feather Safety Razor Co., Ltd., Osaka, Japan). Muscle samples (60 ~ 80 mg) were obtained at 5 cm depth of the middle gluteal muscle using 13-gauge x 3.9 cm co-axial introducer needle and 14-gauge x 9 cm biopsy needle (SuperCore Biopsy Instrument, Argon Medical Devices, Plano, Texas, USA) before, immediately after, 4 h after, and 24 h after the exercise. All muscle samples were immediately frozen in liquid nitrogen and stored at −80°C until analyzed.

### 2.6. Biochemical analysis

The muscle pieces (~20 mg) were exposed to 2 M perchloric acid at −20°C for 20 min to extract glycogen. The samples were then neutralized by 2 M KHCO_3_. Glycogen content was measured in perchloric acid-denatured protein pellets. To hydrolyze glycogen, the pellets were boiled in 2 N HCl for 2 h at 100°C and then neutralized with 2 N NaOH. The glycogen content was determined with spectrophotometric techniques according to procedures previously described (33).

### 2.7. Real-time RT-PCR

The mRNA expression of PGC-1α, succinate dehydrogenase (SDH), VEGF, angiopoietin 1 (ANGPT1), hypoxia Inducible Factor 1-α (HIF1-α), phosphofructokinase (PFK), and monocarboxylate transporter (MCT) 1 and 4 were determined as described previously (10, 22). Total RNA was extracted from each muscle sample (~20 mg) with TRIZOL reagent (Molecular Probes, Breda, Netherlands). The purity and quantity of total RNA were determined by measuring the absorbance of aliquots at 260 and 280 nm. Total RNA was then treated for 30 min at 37°C with TURBO DNase (Ambion, Austin, TX) to remove genomic dNa from samples. dNase- treated RNA (0.5 μg) was used to synthesize cDNA with an Exscript™ RT reagent Kit (Takara, Tokyo, Japan). Thereafter, the cDNA products were analyzed by real-time PCR using the SyBr Green PCR Master Mix protocol in a Stepone™ real Time PCR System (Applied Biosystems Japan, Tokyo, Japan). The amplification program included an initial denaturation step at 95°C for 10 min, followed by 40 cycles of denaturation at 95°C for 30 s, and annealing/extension at 58°C for 1 min. The amount of glyceraldehyde-3-phosphate dehydrogenase (GAPDH) mRNA was estimated as an internal control. Each mRNA was normalized to GAPDH by subtracting the cycle threshold (ct) value of GAPDH from the Ct value of the gene target [ΔCt (target)]. The relative expression of the target gene was calculated as the relative quantification value for the pre value. Following the relative expression, dissociation-curve analysis detected no non-specific amplification in cDNA samples. The sequences of the specific primers used in this study were presented in Table 1. Each PCR primer was designed by primer express® software (Applied Biosystems Japan), and the oligonucleotides were purchased from FASMAC (FASMAC, Kanagawa, Japan).

**Table 1:** The sequences of the specific primers for real-time RT-PCR.

	Forward sequence	Reverse sequence
GAPDH	CAAGGCTGTGGGCAAGGT	GGAAGGCCATGCCAGTGA
PGC-1α	TCCGTGTCACCACCCAAAT	TGAACGAGAGCGCATCCTT
SDH	AGGTTTGCTGATGGCAGTATAAGA	TGCATCGACTTCTGCATGCT
VEGF	CCCACTGCGGAGTTCAACAT	TTGGCTTTGGTGAGGTTTGAT
ANGPT1	GCAAATGTGCCCTCATGCT	CAGATTGGATGGGCCACAAG
HIF1-α	AAGTGCGAGCACGATTACAGTATT	GACGGTAGGAAGAGCAGGTTCTT
CD34	CCGCGCTCTGCTTGCT	GCAGTCGAGTTTTCCTCTGTGA
PFK	GGTGGCACAGTGATTGGAAGT	CGGAGTCGTCCCTCTCGTT
MCT1	GATTCTTGGCGGCTGCTTGTCAGG	TGCCAATCATGGTCAGAGCCGGA
MCT4	ATGGTGTCTGCGTCCTTCTGCGGA	AGCGCCAAACCCAAGCCGGTAA

GAPDH, glyceraldehyde 3-phosphate dehydrogenase; PGC-1α, peroxisome proliferator-activated receptor γ co-activator-1α; SDH, succinate dehydrogenase complex; VEGF, vascular endothelial growth factor; ANGPT1, angiopoietin 1; HIF1-α: hypoxia Inducible Factor 1-α; PFK, phosphofructokinase; MCT1, monocarboxylate transporter 1; MCT4, monocarboxylate transporter 4.

### 2.8. Western blotting

The procedure for western blotting was described previously (34). Briefly, gluteus medius muscle samples (~20 mg) were homogenized in lysis buffer (25 mmol/L Tris–HCl, pH 7.6, 150 mmol/L NaCl, 1% NP-40, 1% sodium deoxycholate, and 0.1% sodium dodecyl sulfate [SDS]) supplemented with protease inhibitor mixture (Complete Mini, ETDA-free, Roche Applied Science, Indianapolis, IN) and phosphatase inhibitor mixture (PhosSTOP, Roche Applied Science). The total protein content of samples was quantified using the Quick protein assay (Bio-Rad). Equal amounts of protein (10–15 μg) were loaded onto 10% SDS-PAGE gels and separated by electrophoresis. Proteins were transferred to polyvinylidene difluoride membranes, and blotting was carried out. Commercially available antibodies were used to detect total AMPKα, phospho-AMPKα (Thr172), total p38 MAPK, and phospho-p38 MAPK (Thr180/Tyr182) (Table 2). Blots were scanned using commercial imaging system (ChemiDoc XRS+, Bio-Rad) and band intensities were quantified using commercial software (Image Lab 5.2.1, Bio-Rad). Phosphorylation of AMPK and p38 MAPK was corrected to their respective total protein content and was also normalized to pre-exercise value.

**Table 2:** The primary antibodies for western blotting.

Primary antibodies	Manufacturer and product number	RRID
AMPKα	Cell Signaling Technology, #2532	AB_330331
Phosphorylated AMPKα (Thr172)	Cell Signaling Technology, #2531	AB_330330
p38 MAPK	Cell Signaling Technology, #9212	AB_330713
Phosphorylated p38 MAPK (Thr180/Tyr182)	Cell Signaling Technology, #9211	AB_331641

AMPKα, AMP-activated protein kinase; p38 MAPK, p38 mitogen-activated protein kinase.

### 2.9. Statistical analysis

Data are presented as mean ± standard error (SE). The Shapiro–Wilk test was applied to test the normality of data distributions. Heart rate and blood gas variables were analyzed using mixed models with exercise protocol as a fixed effect and horse as a random effect. Glycogen content, phosphorylation of proteins, and mRNA expressions were analyzed using mixed models with exercise protocol, time, and exercise protocol x time interaction as fixed effects and horse as a random effect. Tukey’s tests were used for multiple comparison. Statistical analyses were performed using commercial software (JMP 16.1.0, SAS Institute Inc., Cary, NC) with significance defined as *p* < 0.05.

## 3. Results

### 3.1. Heart rate and blood gas variables during each exercise

**Figure 1:**
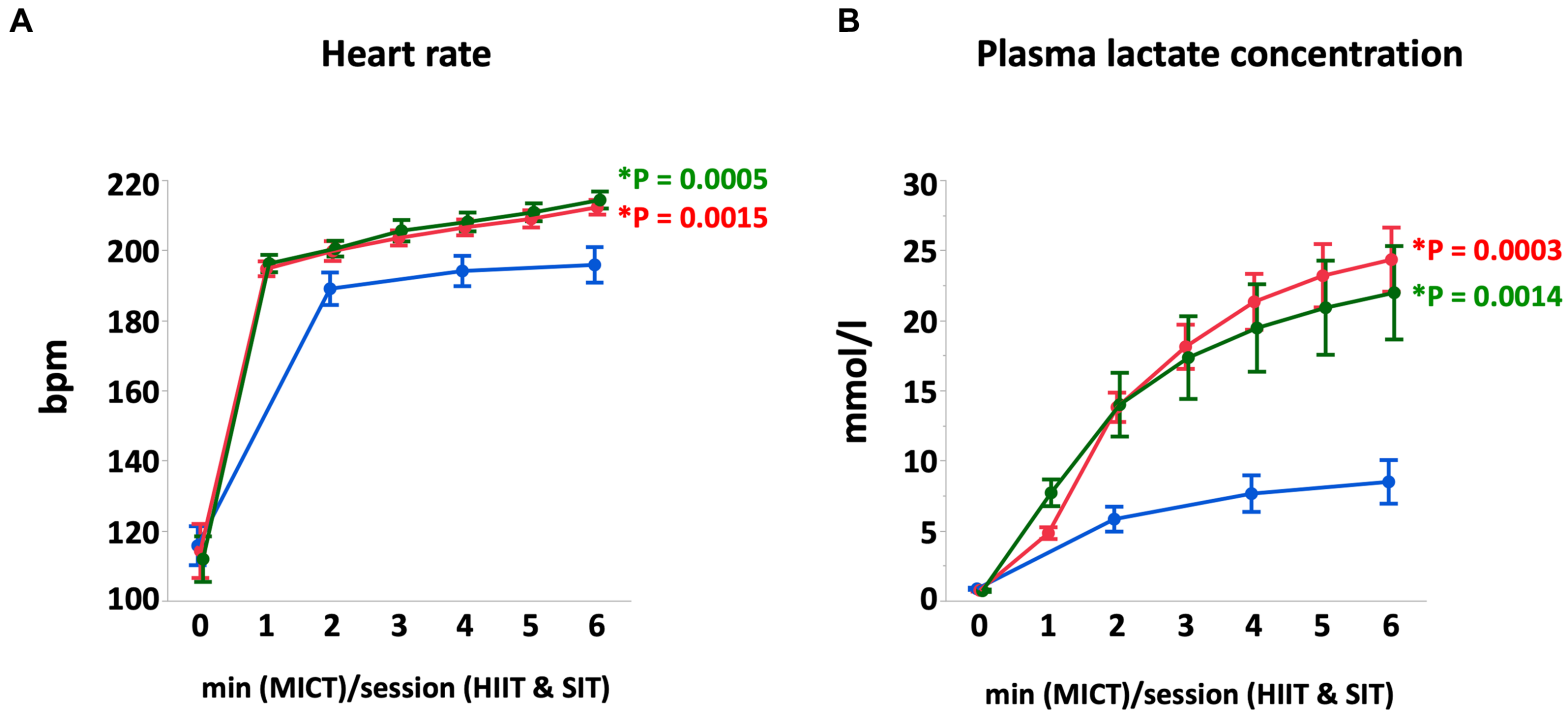
Heart rate **(A)** and plasma lactate concentration **(B)** during MICT, HIIT, and SIT. Values are mean ± SE (*n* = 8). *Significant difference vs. MICT at the end of exercise (*p* < 0.05).

**Figure 2:**
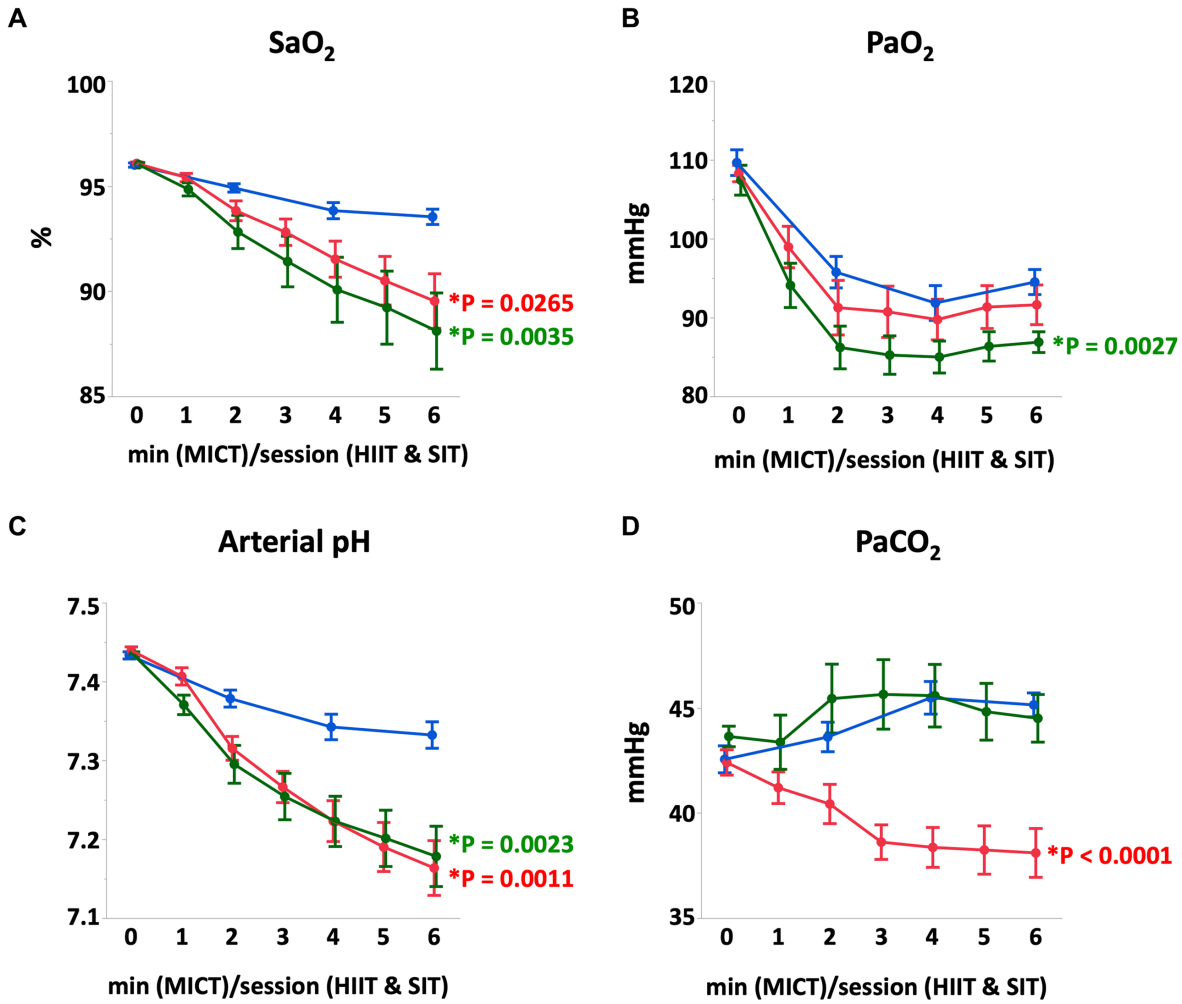
Arterial O_2_ saturation **(A)**, arterial O_2_ partial-pressure **(B)**, arterial pH **(C)**, and arterial CO_2_ partial-pressure **(D)** during MICT, HIIT, and SIT. Values are mean ± SE (*n* = 8). *Significant difference vs. MICT at the end of exercise (*p* < 0.05).

At the end of each exercise protocol, mean heart rate in HIIT and SIT were higher than that in MICT (HIIT vs. MICT, *p* = 0.0005; SIT vs. MICT, *p* = 0.0015; Figure 1). Plasma lactate concentrations increased gradually during each exercise and reached 22.0 ± 3.3 mmoL/L in HIIT and 24.3 ± 2.3 mmoL/L in SIT at the end of exercise, which were higher compared with that in MICT (8.5 ± 1.6 mmoL/L; HIIT vs. MICT, *p* = 0.0014; SIT vs. MICT, *p* = 0.0003; Figure 1). At the end of exercise, arterial O_2_ saturation (SaO_2_) and arterial pH in HIIT and SIT were significantly lower than those in MICT (SaO_2_, HIIT vs. MICT, *p* = 0.0035; SIT vs. MICT, *p* = 0.0265; pH, HIIT vs. MICT, *p* = 0.0011; SIT vs. MICT, *p* = 0.0023; Figure 2). Arterial O_2_ partial pressure (PaO_2_) in HIIT was lower than that in MICT (*p* = 0.0027) and arterial CO_2_ partial pressure (PaCO_2_) in MICT and HIIT were higher than that in SIT (MICT vs. SIT, *p* < 0.0001; HIIT vs. SIT, *p* < 0.0001; Figure 2). Pulmonary artery temperature (T_PA_) increased linearly in all groups and, at the end of exercise, T_PA_ in HIIT (41.3 ± 0.2°C) was higher than that in MICT (40.4 ± 0.2°C, *p* = 0.0034), but T_PA_ in SIT (40.9 ± 0.2°C) was not significantly different from that in MICT and HIIT.

### 3.2. Muscle glycogen

**Figure 3:**
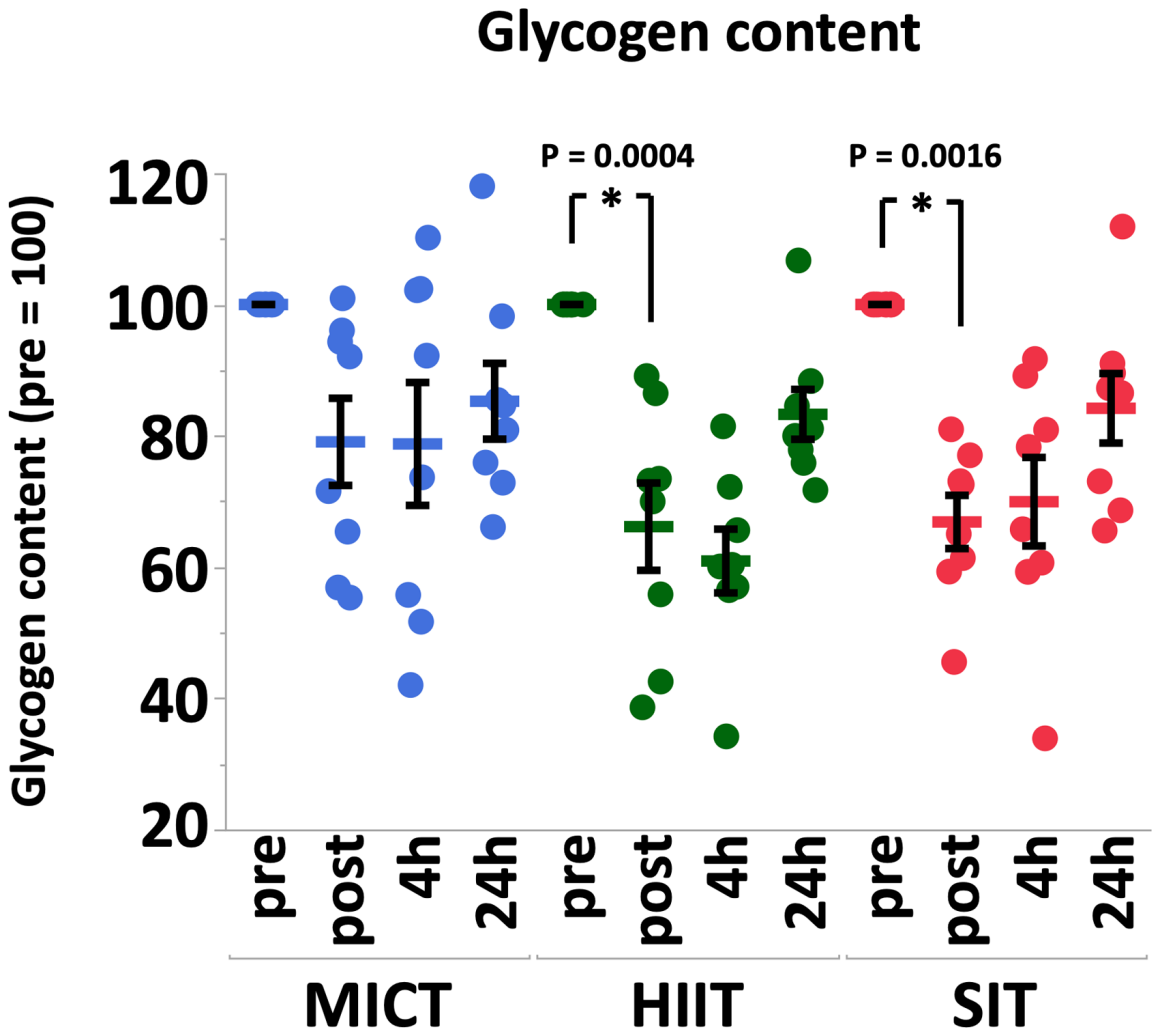
Muscle glycogen contents before (pre), immediately after (post), 4 h after (4 h), and 24 h after (24 h) MICT, HIIT, and SIT. Values are mean ± SE (*n* = 8). *Significant difference from pre (*p* < 0.05).

Muscle glycogen content decreased significantly immediately after exercise in HIIT (*p* = 0.0004) and SIT (*p* = 0.0016) immediately after exercise, but not in MICT (*p* = 0.19; Figure 3). After 4 h of recovery, muscle glycogen content in all groups remained at similar levels compared with post-exercise values, but progressively recovered at 24 h after exercise (Figure 3).

### 3.3. Phosphorylation of AMPK and p38 MAPK

**Figure 4:**
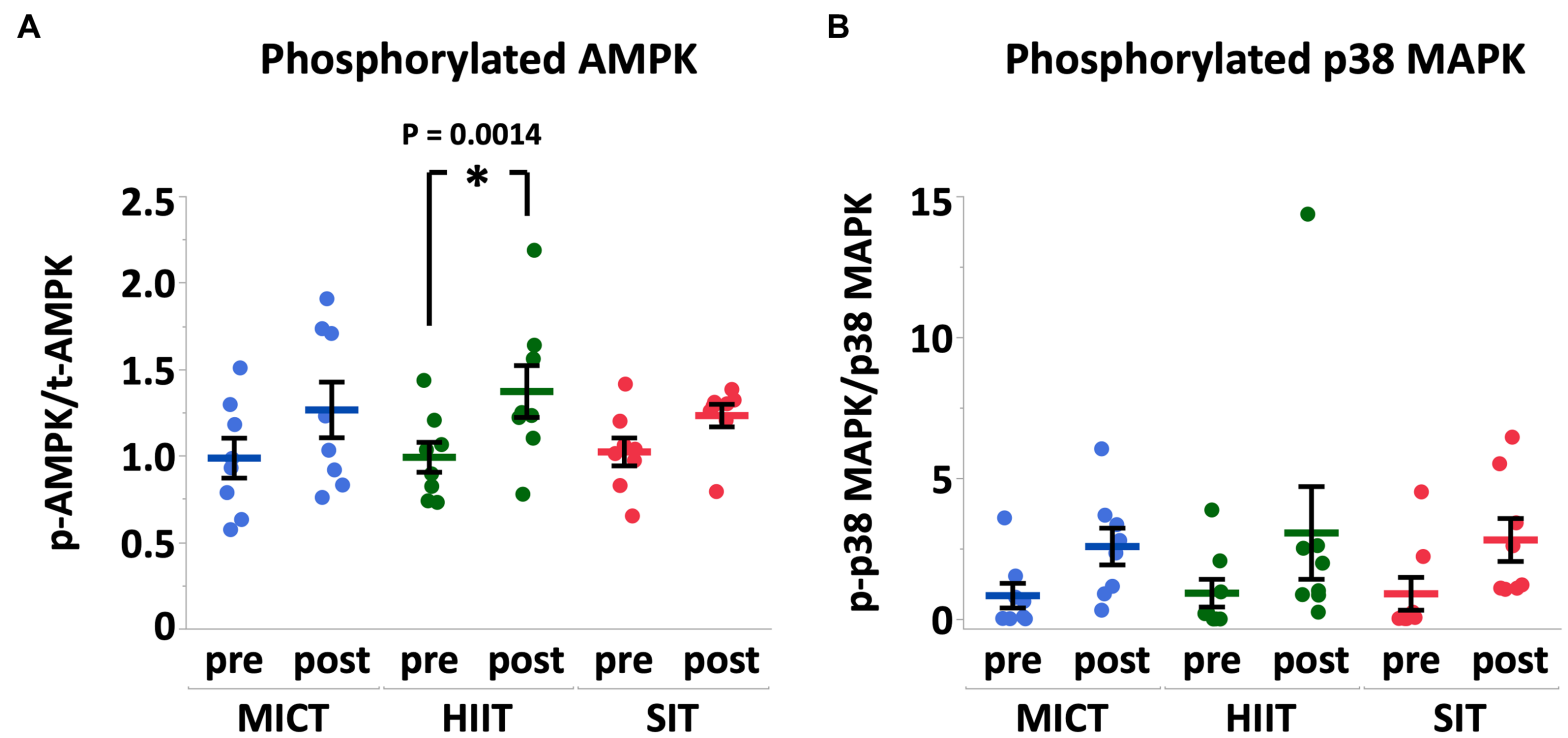
Phosphorylation of AMPKα **(A)** and p38 MAPK **(B)** expressed relative to total AMPKα and total p38 MAPK, respectively, before (pre) and immediately after (post) MICT, HIIT, and SIT. Pre values are normalized to 1. Values are mean ± SE (n = 8). *Significant difference from pre (*p* < 0.05).

Immediately after exercise, total AMPKα (MICT, 0.85 ± 0.12 fold, *p* = 0.84; HIIT, 0.84 ± 0.08 fold, *p* = 0.81; SIT, 0.96 ± 0.14 fold, *p* = 1.00) and total p38 MAPK protein did not change in all groups (MICT, 1.00 ± 0.08 fold, *p* = 0.99; HIIT, 0.91 ± 0.09 fold, *p* = 0.99; SIT, 0.91 ± 0.10 fold, *p* = 0.81). The phosphorylation of AMPKα protein significantly increased in HIIT (*p* = 0.014) compared to pre-exercise, whereas the phosphorylation of p38 MAPK protein did not change significantly in all groups (MICT, *p* = 0.66; HIIT, *p* = 0.45; SIT, *p* = 0.57) (Figure 4).

### 3.4. Exercise-induced mRNA responses

**Figure 5:**
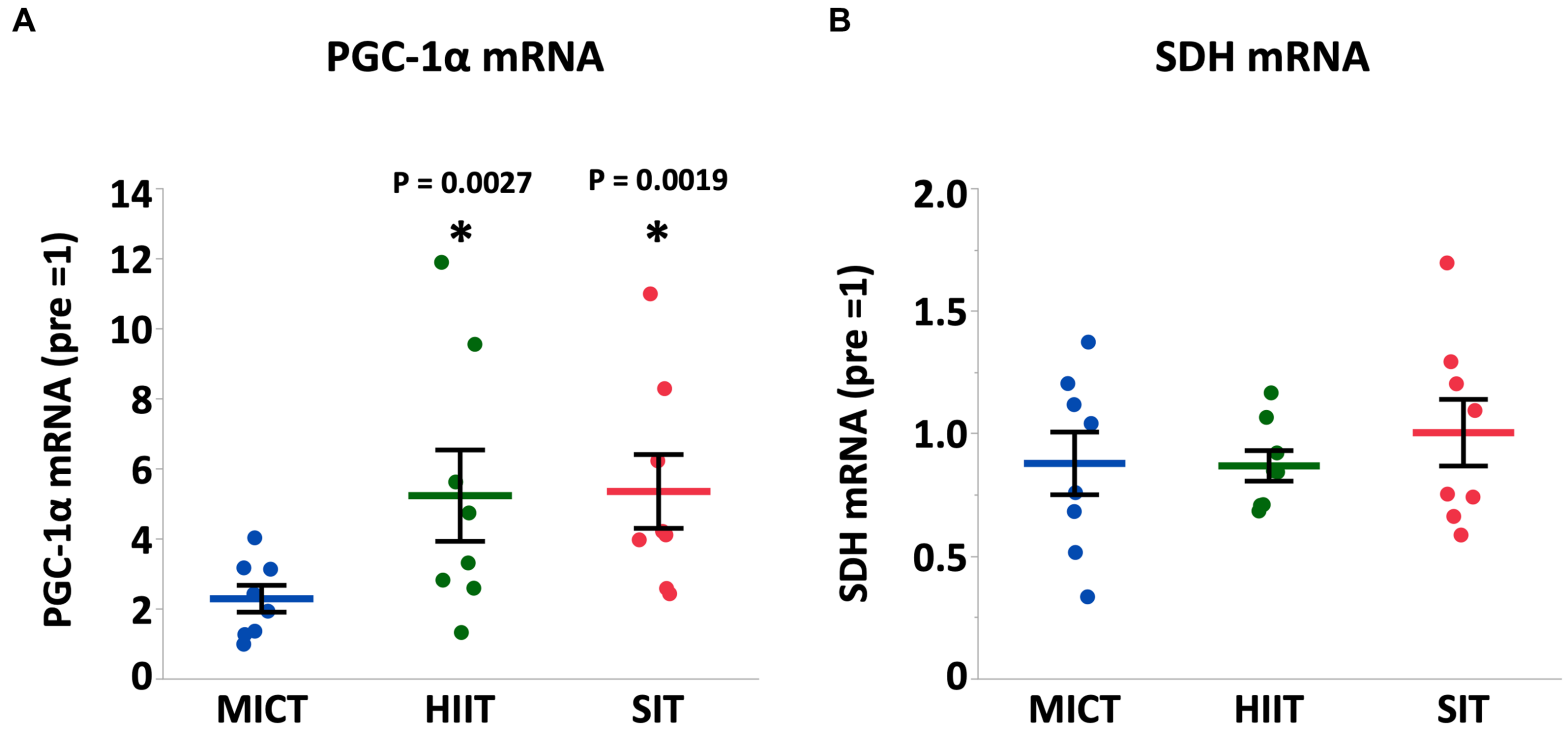
PGC-1α **(A)** and SDH **(B)** mRNA expression at 4 h after (4 h) each session of MICT, HIIT, and SIT. Values are normalized to GAPDH mRNA expression and are expressed relative to pre. Values are mean ± SE (*n* = 8). *Significant difference from pre (*p* < 0.05). †Significant difference between exercise protocols (*p* < 0.05).

**Figure 6:**
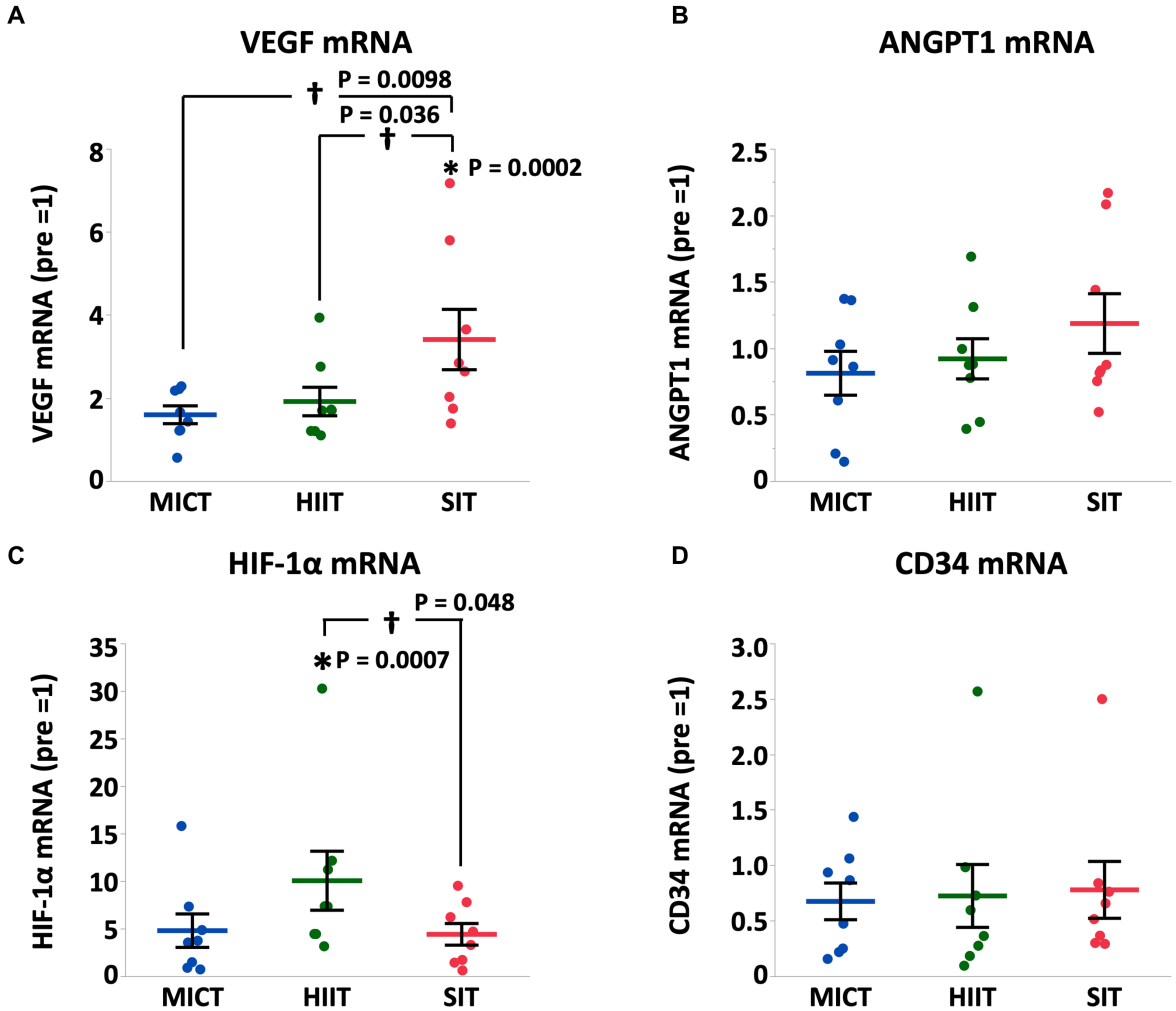
VEGF **(A)**, ANGPT1 **(B)**, HIF-1α **(C)**, and CD34 **(D)** mRNA expression at 4 h after (4 h) each session of MICT, HIIT, and SIT. Values are normalized to GAPDH mRNA expression and are expressed relative to pre. Values are mean ± SE (*n* = 8). *Significant difference from pre (*p* < 0.05). †Significant difference between exercise protocols (*p* < 0.05).

**Figure 7:**
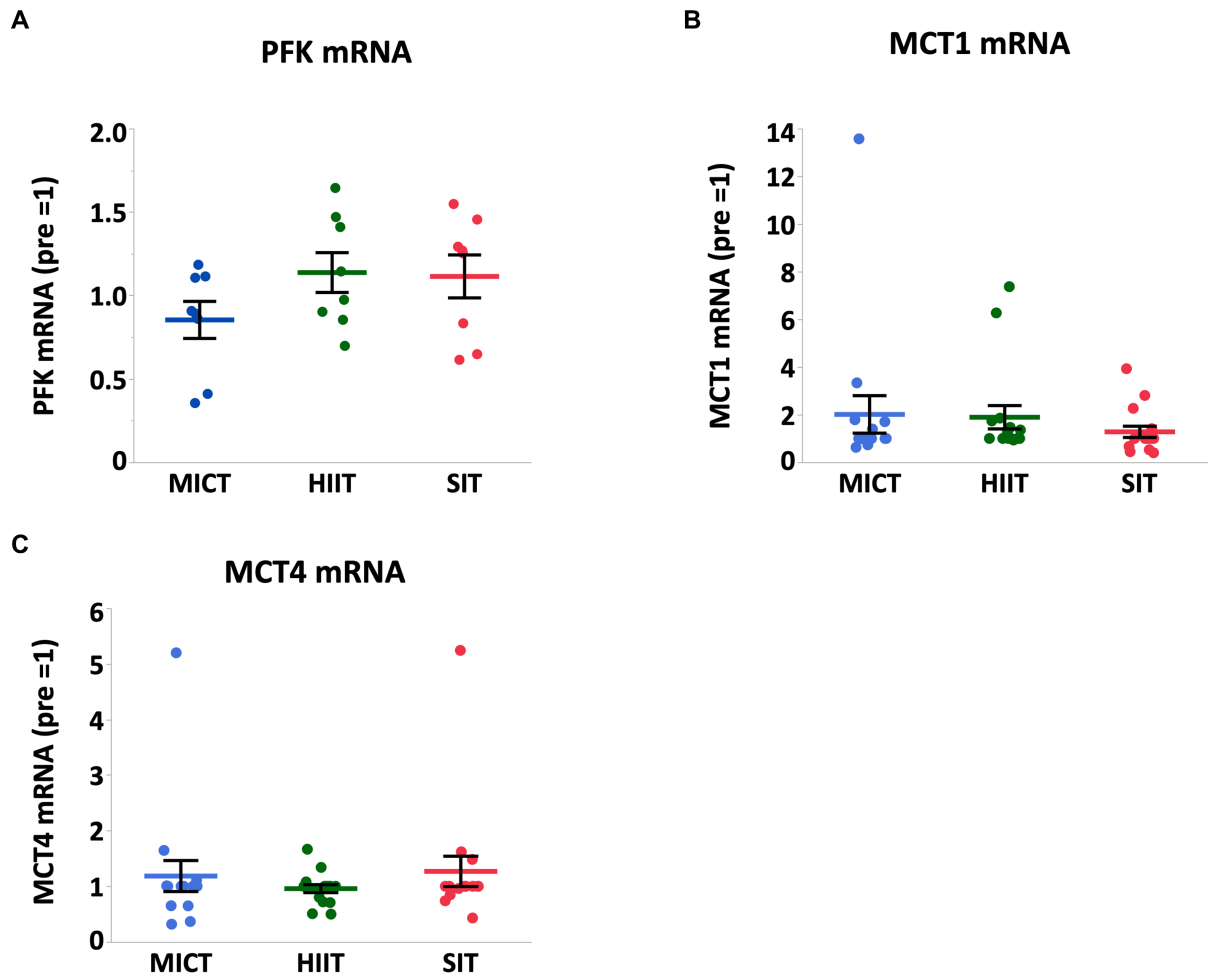
PFK **(A)**, MCT1 **(B)**, and MCT4 **(C)** mRNA expression at 4 h after (4 h) each session of MICT, HIIT, and SIT. Values are normalized to GAPDH mRNA expression and expressed relative to pre. Values are mean ± SE (*n* = 8). *Significant difference from pre (*p* < 0.05). †Significant difference between exercise protocols (*p* < 0.05).

At 4 h after each exercise, PGC-1α mRNA increased in HIIT and SIT compared to pre-exercise (HIIT, *p* = 0.0027; SIT, *p* = 0.0019) with tendencies to be higher than MICT (MICT vs. HIIT, *p* = 0.062; MICT vs. SIT, *p* = 0.067; Figure 5). VEGF mRNA increased in SIT (*p* = 0.0002) with significant differences between SIT and the other two groups (MICT vs. SIT, *p* = 0.0098; HIIT vs. SIT, *p* = 0.036; Figure 6). HIF-1α mRNA increased in HIIT (*p* = 0.0007) with a significant difference between HIIT and SIT (*p* = 0.048; Figure 6). There were no significant mRNA changes at 4 h after exercise in SDH (MICT, *p* = 0.88; HIIT, *p* = 0.85; SIT, *p* = 1.00) (Figure 5), ANGPT1 (MICT, *p* = 0.90; HIIT, *p* = 1.00; SIT, *p* = 0.91), CD34 (MICT, *p* = 0.73; HIIT, *p* = 0.84; SIT, *p* = 0.93)(Figure 6), PFK (MICT, *p* = 0.81; HIIT, *p* = 0.85; SIT, *p* = 0.93), MCT1 (MICT, *p* = 0.45; HIIT, *p* = 0.58; SIT, *p* = 1.00), and MCT4 (MICT, *p* = 0.96; HIIT, *p* = 1.00; SIT, *p* = 0.85) (Figure 7).

## 4. Discussion

Our results show that HIIT and SIT induced more severe metabolic acidosis and hypoxemia compared with MICT despite the same run distance. In skeletal muscle, we provide the findings that (1) both HIIT and SIT utilized glycogen to a greater extent compared with MICT, (2) HIIT induced the phosphorylation of AMPK and the upregulation of PGC-1α and HIF-1α mRNA, and (3) SIT upregulated PGC-1α and VEGF mRNA to a greater extent compared with MICT. These findings suggest that HIIT and SIT induce greater physiological and skeletal muscle responses compared to MICT when exercise volumes are equal.

### 4.1. HIIT and SIT induced more severe metabolic acidosis and hypoxemia

Heart rate increases proportionally as exercise Intensity increases and reaches a plateau at maximal heart rate, so it is reasonable that heart rate in MICT was lower than those of HIIT and SIT with similar values in HIIT and SIT. Plasma lactate concentration increases exponentially as exercise intensity increases, which is different from heart rate. Plasma lactate concentration and arterial pH are closely related and both indicate the increase of glycolysis and H^+^ production during intense exercise (35). As expected, plasma lactate concentration in MICT was lower than those in HIIT and SIT, and arterial pH in MICT was higher than those in HIIT and SIT. However, there were no statistical differences between HIIT and SIT both in plasma lactate concentration and arterial pH. The exercise intensity was higher in SIT compared to HIIT (120% VO_2_max vs. 100% VO_2_max), whereas exercise duration was shorter in SIT than that in HIIT (15 s × 6 vs. 30 s × 6). Plasma lactate and arterial pH are affected by both intensity and duration of exercise and this could be the reason why there were no differences between HIIT and SIT in spite of SIT having greater intensity and HIIT having longer duration.

Thoroughbred horses often experience arterial hypoxemia during intense exercise, and exercise-induced hypoxemia is reported to be caused mostly by a limitation in diffusion in the lungs (36). In this study, we observed more severe hypoxemia in HIIT and SIT compared with MICT as expected. Similar to lactate concentration and pH, the exercise intensity in SIT was higher than that in HIIT, but the exercise duration in SIT was half of that in HIIT, counteracting the intensity and duration of exercise.

Metabolic and hypoxic stimulus during acute exercise induces physiological adaptation and the extent of these adaptation depends on various factors, including the intensity, duration, mode of exercise, training status, and trainability of subject individuals (11). Among these factors, exercise intensity is considered to be the prominent factor that regulates exercise-induced physiological adaptation (1, 37). In this study, HIIT and SIT induced more severe metabolic acidosis and hypoxemia compared to MICT, as we hypothesized.

### 4.2. Responses in mitochondrial biogenesis after exercise

AMPK and p38 MAPK are two major regulators linked to PGC-1α and the signaling pathway of mitochondrial biogenesis in skeletal muscle (38, 39). During moderate to intense exercise, muscle glycogen is the main carbohydrate source and the rate of glycogen degradation and recruitment of muscle fibers are dependent on the intensity, duration, and mode of exercise. Given that the central role for AMPK is regulating intracellular energy metabolism in response to acute energy depletion, it is reasonable that we found a significant activation of AMPK immediately after HIIT that also showed a significant decrease in muscle glycogen content (−34%). While SIT showed a similar decrease in muscle glycogen content (−33%), the change in the phosphorylation of AMPK was not significant in SIT. AMP and ADP bind to the binding site on the AMPK regulatory domain and protect AMPK from dephosphorylation. Therefore, an increase in ADP during exercise may be the primary signal that promotes increased phosphorylation of AMPK (40). The difference in the recovery period between HIIT and SIT (30 s vs. 75 s) may cause a variation in the AMP:ATP ratio during exercise and may induce a greater activation of AMPK in HIIT. The p38 MAPK is sensitive to mechanical stress and phosphorylation of p38 MAPK is increased after both endurance exercise (41) and sprint interval exercise (20). In this study, we observed a > 3-fold increase in phosphorylation of p38 MAPK after all exercise protocols, but the increases were not statistically significant. Phosphorylation of p38 MAPK is closely related to muscle contraction, and we hypothesized that HIIT and SIT induce a greater activation of p38 MAPK compare to MICT. However, variations in the results of western blotting seemed be considerably large in phosphorylated p38 MAPK.

In mitochondrial biogenesis, PGC-1α is the most important regulator and coordinates several regulatory factor cascades and activates transcription factors such as mitochondrial transcription factor A and nuclear respiratory factors (41, 42). In this study, PGC-1α mRNA was significantly increased after 4 h in HIIT and SIT, but not in MICT, suggesting that HIIT and SIT are likely to be superior to MICT for promoting PGC-1α abundance and mitochondrial biogenesis. Several human studies have demonstrated that the exercise-induced increase in PGC-1α mRNA depends on exercise intensity (43, 44). Considering the evidence in previous human research, higher exercise intensities in HIIT and SIT, even with intermittent recovery periods, appear to be the major factor that regulates the increase in PGC-1α mRNA in the present study. Combes et al. demonstrated that a single session of intermittent exercise (30 × 1-min at 70% VO_2_max) induces a greater activation of AMPK and p38 MAPK when compared to continuous exercise (30 min at 70% VO_2_max) of matched work and intensity (45). However, it is difficult to distinguish the effect of exercise intensity from that of exercise mode in this study design because we did not use high-intensity continuous exercise. Perry et al. reported that the mRNA response of PGC-1α after each HIIT session is attenuated as training progressed despite a continual increase in training power output (16). Therefore, it remains unclear whether the greater mRNA response in HIIT and SIT is maintained throughout the long-term training period (i.e., weeks or months) and further research is required to determine the effect of intermittent modality and chronic training.

In contrast, SDH mRNA did not change following exercise with all protocols and was not different between exercise protocols in this study. Previous research suggests that the initial increases in mRNA encoding mitochondrial oxidative enzymes (CS and COX IV) were more delayed after the training session compared to the mRNA encoding some of the transcription proteins (PGC-1α, PGC-1β, and PPAR) (16, 46) and the similar late response might occur with SDH. Furthermore, mitochondrial content has been reported to correlate with training volume, but not with training intensity (47). Also, it has been suggested that training intensity modulates change in PGC-1α protein content and mitochondrial respiration, but not in markers of mitochondrial content in human skeletal muscle (48). Therefore, our results are, at least partly, reasonable because exercise volumes were equal in all exercise protocols, and exercise volume itself (6 min at 70% VO_2_max, 6 × 30 s at 100% VO_2_max and 6 × 15 s at 120% VO_2_max) may not be enough to stimulate SDH mRNA expression in horses.

### 4.3. Responses in angiogenesis after exercise

Angiogenesis is the branching of new blood vessels from existing blood vessels and is regulated by several angiogenic factors including VEGF and HIF-1α with VEGF playing a central role in its regulation (49). Exercise induces a variety of adaptations, including upregulation of angiogenesis, which, in turn, contributes to exercise adaptation (50). Several studies have evaluated changes associated with post-exercise angiogenesis and have shown that high intensity exercise induces angiogenesis (21). In this study, VEGF mRNA in SIT showed a significant increase and significant differences compared to that in MICT and HIIT at 4 h after exercise, which is consistent in that exercise intensity was the highest in SIT among the 3 exercise protocols.

HIF-1α is a transcriptional factor that regulates VEGF expression in response to hypoxia (17). HIF-1α regulates the pre-angiogenic activity of VEGF, which regulates the expression of many genes and induces angiogenesis (51). We previously reported that 4 weeks of high-intensity training (100%VO_2_max for 2 min, 3 days/week) in hypoxia (F_I_O_2_ = 0.15) induces upregulation of VEGF mRNA and significant increases in VO_2_max and capillary density (10). In this study, SaO_2_ and PaO_2_ in HIIT were the lowest among the 3 exercise protocols and it is reasonable that the most severe hypoxemia in HIIT stimulated HIF-1α pathway and showed a higher HIF-1α mRNA expression at 4 h after exercise. However, the increased HIF-1α mRNA in HIIT did not induce a significant increase in VEGF mRNA in this study, possibly because exercise intensity may be a more predominant factor in angiogenesis compared with hypoxemia and because exercise-induced hypoxemia during HIIT was not strong enough to augment VEGF mRNA. There were no significant increases in angiogenesis-related gene expression after MICT in this study. The exercise protocol for MICT did not cause hypoxemia and may not have been sufficient to stimulate the angiogenetic pathway in horses. Collectively, HIIT and, especially, SIT seem to promote angiogenesis to a greater extent than MICT. Oxygen delivery to mitochondria via capillaries in the skeletal muscle is an important factor in aerobic capacity. Therefore, HIIT and SIT are considered to be more suitable exercise modes for improving aerobic exercise capacity compared with MICT.

### 4.4. Lactate transporters in skeletal muscle

Lactate is produced mainly in fast-twitch muscle fibers during high-intensity exercise and is transported to slow-twitch muscle fibers via monocarboxylate transporter (MCT) (52). MCT1 is located mostly in slow oxidative fibers and transports lactate into muscle cells to utilize as a substrate, while MCT4 is located in fast glycolytic muscle and transports lactate out of muscle cells (53). Our previous study found that MCT1 protein content correlates with CS activity in equine skeletal muscle and MCT4 protein content also correlates with the increase in plasma lactate concentration at the IET (54), suggesting that lactate transport and utilization during exercise is closely related to substrate oxidation, lactate extraction and maximal exercise performance. In addition, recent studies have reported that acute lactate administration increases PGC-1α mRNA expression in mouse skeletal muscle (55) and 3 weeks of lactate administration increases mitochondrial enzyme (CS and COX) activity in mice (56). These results suggests that lactate is not only substrate but also works as a signaling molecule that regulates exercise-induced adaptations such as mitochondrial biogenesis. We have reported that 18 weeks of high-intensity training (90–110% VO_2_max for 3 min, 5 days/week) increased MCT1 and MCT4 protein expression, and the following 6 weeks of moderate-intensity training (70%VO_2_max for 3 min, 5 days/week) maintained MCT1 protein, but not MCT4 protein in horses (54). In contrast to our expectation, there were no significant increases in MCT1 and MCT4 mRNAs after exercise in all exercise protocols including HIIT and SIT. We assumed that plasma lactate concentrations in HIIT (22.0 ± 3.3 mmoL/L) and SIT (24.3 ± 2.3 mmoL/L) were high enough to stimulate gene expressions of both MCT1 and MCT4. Our previous study demonstrated that MCT1 and MCT4 mRNA expressions were increased at 6 h after the IET in trained horses (57). The run distance at the IET in the previous study was 4,901 ± 302 m (57), whereas the run distance in this study was approximately 2,890 ± 44 m (2000 m shorter than the previous study). Therefore, the exercise volume in the present exercise protocols may be insufficient to upregulate MCT1 and 4 mRNA. In addition, in rat white gastrocnemius muscle, MCT1 mRNA transiently increased immediately after 2 h of treadmill and decreased to baseline at 5 h and 10 h after exercise, and increased again at 24 h after exercise, while MCT4 mRNA did not change until 24 h after exercise (58). Given that nearly 90% of the middle gluteal muscle in Thoroughbred horses are fast-twitch fibers similar to rat white gastrocnemius muscle, the similar results might occur in our study. Furthermore, according to the results of a human study that investigated the time-course changes in MCT protein and mRNA over 72 h after a final session of 4 weeks of HIIT, both MCT1 and MCT4 mRNA did not change at 3 h and 9 h after exercise and progressively decreased at 24 h and 72 h relative to week 0 (59). Collectively, the sampling timing in this study (4 h after exercise) may not be appropriate for evaluating the gene expression of MCTs.

In conclusion, HIIT and SIT caused more severe arterial hypoxemia and lactic acidosis compared with MICT despite the equal run distance. In addition, HIIT activated the AMPK signaling cascade, and both HIIT and SIT elevated mitochondrial biogenesis and angiogenesis in skeletal muscle, whereas MICT did not induce any significant changes to these signaling pathways. Taken together, high-intensity interval and sprint interval exercise induce greater physiological and skeletal muscle responses compared to moderate-intensity continuous exercise when exercise volumes are equal. Therefore, high-intensity interval training is a potential candidate for a new training strategy for Thoroughbred horses.

## Data availability statement

The original contributions presented in the study are included in the article/supplementary material, further inquiries can be directed to the corresponding author.

## Ethics statement

Exercise protocols for the study were reviewed and approved by the Animal Welfare and Ethics Committee of the Japan Racing Association (JRA) Equine Research Institute (Permit numbers: 2020-10 & 2020-11). The study was conducted in accordance with the local legislation and institutional requirements.

## Author contributions

KM, OH, and HM contributed to conception and design of the study. KM, YT, and YE collected samples. KM, OH, YT, YE, and KY performed experiments. KM and HM organized the database. KM performed the statistical analysis. KM wrote the first draft of the manuscript. All authors contributed to manuscript revision and approved the submitted version.
